# Case of Fracture of Upper and Lower Jaw

**Published:** 1868-06

**Authors:** T. O. Oliver


### ARTICLE III.

### *Case of Fracture of Upper and Lower Jaw.*

By T. O. Oliver.

Luke, O. N.—Coachman, age 28, came to me June 1st, –67, with upper and lower jaw badly fractured. The circumstances attending the case were these: while exercising his employers horse, the animal, a high spirited brute suddenly whirled around thereby drawing the halter from the hands of his custodian, and striking out with his heels in the air, encountered his keeper with his hoof over the left eye and mouth, fracturing the lower jaw in five, and the upper in four places. The fracture of the lower jaw commenced on the right side at the second bicuspid, crushing over to the molar on the left, bisecting the eye tooth, carrying away the entire crown of the lateral incisor, and driving the fang of the latter down into the jaw, where it lodged. This being an alveolar fracture, ready adjustment of the parts was difficult and perplexing, as the entire process when first examined, was found lying under the tongue. After placing ligatures around the several separate fractures, and bringing the different parts into juxtaposition, with great difficulty I succeeded in obtaining a good gutta-percha impression, (having failed with wax) which I allowed to remain until the following day. I then adopted in its entirety, the No. 4, interdental splint invented by Doctor Thomas B. Gunning of New York City, removing it semi-weekly. On the tenth day after the accident, the swelling having greatly diminished, I found upon examination considerable congestion, which was overcome by the aid of a couple of leeches. Three weeks from the date of the accident, I took an impression with wax, and made a hard rubber splint—with wings, which the patient continued to wear for the space of three months. At this period finding good union, the wings were abolished, and he wore the splint at meal times only, for the two subsequent months. On the seventh month he masticated with artificial appliances.

*The Upper Jaw.* When the accident occurred, the upper jaw was fractured in several places down to the soft palate. I succeeded in saving the first, and second molars upon either side, first having cut through the lacerated gum and removed numerous bone spicula, by the use of the bone forceps. I then trimmed away the damaged surplus of gum until I obtained the requisite eveness of edges, which, brought together and held in contact by a temporary splint, resulted in union by first intention. After the lapse of eleven months both jaws were perfectly strong and healthy.

I omitted to state that in manipulating the upper jaw I was obliged, in order to obtain an even arch, to cut away seven eights of an inch of the palate, thereby leaving the arch diminished to such an extent and so irregular in outline, that without the aid of the first and second molars, symetrical excellence could not have been obtained.

---

